# The interlink between chrono-nutrition and stunting: current insights and future perspectives

**DOI:** 10.3389/fnut.2023.1303969

**Published:** 2023-12-12

**Authors:** Nurpudji Astuti Taslim, Salsabila Farradisya, William Ben Gunawan, Aulia Alfatihah, Ria Irmelin Br Barus, Liesty Kurnia Ratri, Astri Arnamalia, Hero Barazani, Mrinal Samtiya, Nelly Mayulu, Bonglee Kim, Hardinsyah Hardinsyah, Ervan Surya, Fahrul Nurkolis

**Affiliations:** ^1^Division of Clinical Nutrition, Faculty of Medicine, Department of Nutrition, Hasanuddin University, Makassar, Indonesia; ^2^Medical Programme, Faculty of Medicine Universitas Brawijaya, Malang, Indonesia; ^3^Faculty of Medicine, Alumnus of Department of Nutrition Science, Diponegoro University, Semarang, Indonesia; ^4^Faculty of Health Science, Department of Nutrition Science, Muhammadiyah University of Surakarta, Surakarta, Indonesia; ^5^Faculty of Medicine, Department of Nutrition Science, Diponegoro University, Semarang, Indonesia; ^6^Department of Chemistry, State Islamic University of Sunan Kalijaga (UIN Sunan Kalijaga), Yogyakarta, Indonesia; ^7^Department of Nutrition Biology, Central University of Haryana, Haryana, India; ^8^Faculty of Medicine, Department of Nutrition, Universitas Muhammadiyah Manado, Manado, Indonesia; ^9^Department of Pathology, College of Korean Medicine, Kyung Hee University, Seoul, Republic of Korea; ^10^Senior Professor of Applied Nutrition Division, Faculty of Human Ecology, Department of Community Nutrition, IPB University, Bogor, Indonesia; ^11^Faculty of Medicine, Universitas Indonesia, Jakarta, Indonesia; ^12^Faculty of Sciences and Technology, Department of Biological Sciences, State Islamic University of Sunan Kalijaga (UIN Sunan Kalijaga), Yogyakarta, Indonesia

**Keywords:** chrono-nutrition, stunting, growth, child, undernutrition, chronic nutrition, 1,000 days of life, pregnancy

## Abstract

Stunting is the one factor that is responsible for the irretrievable damage to children’s mental and physical health. Stunting imitates chronic undernutrition throughout the most extreme critical stages of growth and development of a child in their early life, and due to that stunted child does not completely develop and are too short for their age. Stunting is mainly linked with brain underdevelopment, along with lifelong damaging consequences, comprising weakened mental and learning capacity, deprived performance in school during childhood, and enhanced risks of nutrition linked to chronic long-lasting ailments, such as diabetes, hypertension, diabesity, and obesity in the future. In this review, the authors mainly summarize the latest studies related to chronic nutrition and how it is related to stunting. Optimal nutrition, particularly during pregnancy and the first 24 months of a child’s life, is crucial in preventing stunting. Circadian rhythms play a significant role in maternal and fetal health, affecting outcomes such as premature birth and stunting. Maintaining a balanced diet, avoiding late-night carbohydrate-heavy meals during pregnancy, and promoting breastfeeding align with the body’s biological clock, which can benefit newborns in various ways. Providing dedicated spaces for breastfeeding in public places is important to support infant health.

## Graphical abstract

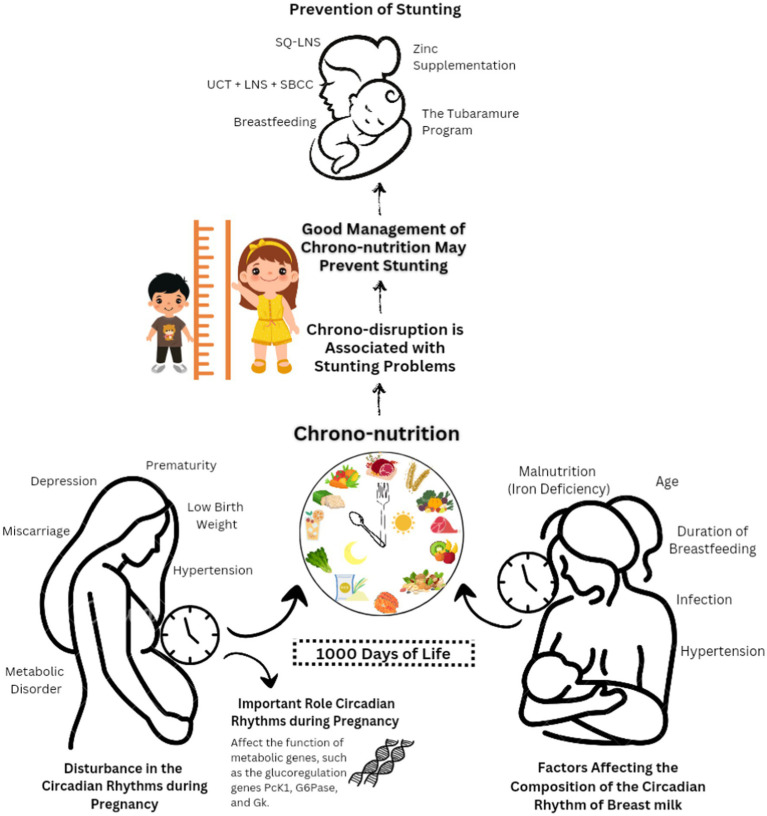

## 1 Introduction

Stunting is a global health problem that occurs most often in low-income and developing countries, like Indonesia. Stunting is characterized by a length or height below the World Health Organization (WHO) standard or a height-for-age z-score less than-2.0 standard deviations. The terms “stunted” or “short infant” describe a toddler whose length-for-age falls below WHO standards (1). From this, it can be concluded that stunted toddlers are definitely stunted, but not necessarily stunted.

According to UNICEF, WHO, and World Bank data and Joint Child Malnutrition Estimates (JME), the prevalence of stunting in young children in 2020 was 22% globally and 27.4% in Southeast Asia. Recently, the global prevalence of stunting has gone down to 21% (2). In 2022, 148.1 million children under 5 years of age worldwide were stunting (3). Based on data from the Indonesian Nutritional Status Survey 2022 published by the Indonesian Ministry of Health, the prevalence of stunting in Indonesia is 21.6% (4). Stunting has short-and long-term effects on child development, including delayed growth and development, which impacts the development of social–emotional skills, such as cognitive skills and performance, decreased physical strength and ability to work, and increased risk of non-communicable diseases, such as diabetes, hypertension, and dyslipidemia (5).

Stunting can be prevented by optimizing the first 1,000 days of life (TF1000DL), starting with meeting nutritional needs from conception to 24 months (6). Optimized nutrition during TF1000DL supports fetal development, keeps the mother healthy in preparation for birth and breastfeeding, and prepares the infant and toddler for optimal growth and development (7). Chrono-nutrition describes the interaction between nutrition and biological rhythms that is relevant to human health. Chrono-nutrition includes energy distribution and meal frequency, regularity, and duration, which have important implications for metabolic health and chronic disease risk (8).

Chrono-nutrition during breastfeeding is essential for improving infant health, ensuring proper development, and promoting cognitive and behavioral neurodevelopment (9). A fetus is exposed to its mother’s circadian rhythms in the womb, including body temperature, food intake, and melatonin levels. Previous research has suggested that circadian rhythms play an important role in a successful pregnancy and optimal fetal development and can reduce the risk of postnatal illness (10). Therefore, this study aims to complement the current research on the role of chrono-nutrition in preventing growth retardation.

## 2 Pathophysiology, risk factors, and consequences of stunting

### 2.1 The first 1,000 days of life and stunting

Human development has a critical phase, namely TF1000DL, which is divided into two parts covering the gestation period and the first 2 years of life. These phases influence a person’s health and ability to function. Even though it has been argued that “catching up” with growth and recovering from stunting may be possible to a certain degree (1), this does not imply that TF1000DL can be neglected. TF1000DL are vulnerable because multiple organs, including the brain, bone, muscle, and fat, grow and develop very quickly (5, 11). Optimal nutrition in TF1000DL supports critical stages of fetal development and maternal health (including postpartum and breastfeeding), and it fosters the growth of infants and toddlers until 2 years of age (6). Evidence suggests that stunting is usually irreversible after TF1000DL, resulting in an intergenerational cycle of poor development in which stunted children continue to be stunted as adults and frequently have stunted offspring (7). Essential nutrients must be provided throughout TF1000DL to prevent developmental delays associated with stunting. However, evidence suggests that stunting in infancy may result in rapid linear growth in childhood and adolescence (i.e., catch-up development). Interestingly, TF1000DL has been considered an insufficient investment in health, and focusing on the first 8,000 days of childhood and adolescence has been suggested (8).

Undernutrition in children is fundamentally caused by insufficient consumption of vitamins and minerals and low-quality or insufficient protein intake (9). Soliman et al. (2021) highlighted the complex role of inactivated mammalian target of rapamycin complex 1 (mTORC1) in the pathogenesis of growth retardation, as it affects lipid and protein metabolism, muscle growth, and immune system autophagy (10, 12). Changes in insulin-like growth factor-1 (IGF-1) also mediate the development of growth retardation (13).

### 2.2 Risk factors and determinants of stunting

Delayed lactation is associated with maternal and intrauterine malnutrition, delayed complementary feeding at >6 months, inadequate (quantity and quality) complementary feeding, and decreased nutritional intake as a result of viral illness (14, 15). Nutritional and health-related issues experienced by expectant mothers have the potential to modify risk profiles and induce alterations in the nutritional and health-related attributes that are imprinted on their offspring, a phenomenon commonly referred to as fetal developmental programming. These alterations can extend their impact to subsequent generations (16). Several studies have summarized risk factors for early childhood stunting, including underweight and short-height mothers, low maternal education, premature birth, short birth length, household environment, economic status, poor food quality, improper feeding techniques, non-exclusive breastfeeding, infections, social and community influences, and child traits (17, 18). Five risk factors for growth retardation have been identified: environmental variables, fetal growth restriction (FGR) and prematurity, fetal nutrition, and maternal and child infection (19). Certain diseases are associated with undernutrition in adulthood (20). In a study, of 1,420 children, 354 (24.93%) were found to be stunted, of which 11.9% were obese compared with 63.17% of normal children. The study reported the impacts of maternal or caregiver literacy on children’s growth (21). The United Nations states that stunting in a child can be influenced by a family’s economic status; toddlers from the lower economic class are more likely to experience stunting (45%) than toddlers from a higher economic class (29%) (22).

### 2.3 Short-and long-term impacts of stunting

Childhood development delay has short-and long-term adverse effects. Stunting, the chronic and severe form of undernutrition characterized by impaired growth and development in children due to inadequate nutrition, has a range of short-term adverse effects. Children affected by stunting often suffer from weakened immune systems, making them more susceptible to infections and illnesses (23). These health issues can lead to frequent bouts of diarrhea and respiratory infections, causing discomfort and distress. Stunted children may also exhibit reduced cognitive abilities and limited learning potential, affecting their performance in school and overall development (24, 25). Additionally, stunting can hinder physical and motor skills, impacting a child’s ability to engage in daily activities and play (26). In sum, the short-term consequences of stunting encompass a compromised immune system, recurrent illnesses, cognitive limitations, and restricted physical development, significantly hampering a child’s well-being and quality of life.

Some of the long-term adverse effects of stunting are increased morbidity and mortality, reduced child growth and learning capacity, increased risk of communicable and non-communicable diseases, and increased susceptibility to adiposity. It reduces fat oxidation and energy expenditure and increases insulin resistance and the risk of developing diabetes, hypertension, dyslipidemia, reduced work capacity, and other undesirable consequences (10). Childhood stunting has significant long-term implications through two mechanisms. First, it directly contributes to reduced adult height and suboptimal functioning in adulthood. Second, it serves as a critical indicator of the early-life processes that give rise to inadequate growth and other unfavorable outcomes (20). Furthermore, research demonstrates that nutritional stunting results in enduring alterations, including decreased energy expenditure, heightened vulnerability to high-fat diets, reduced fat metabolism, and impaired control of food consumption (10). Moreover, stunting has long-term adverse effects on both individuals and society. There is a risk of chronicity when stunting is combined with declining cognitive and academic performance (27), low adult wages, declining productivity, and excessive weight gain in late childhood.

The short and long-term adverse effects of stunting carry significant global and societal implications. Stunting contributes to a substantial global health burden, with millions of children affected worldwide, and its short-term effects, like heightened vulnerability to infections, strain healthcare systems. The long-term health consequences, including a greater risk of chronic diseases in adulthood, further challenge healthcare resources and infrastructure. Economically, the impacts are notable, as short-term consequences such as cognitive limitations can hinder educational attainment, potentially leading to reduced income and economic productivity in adulthood. Societally, stunting-related healthcare costs and reduced workforce productivity can hinder economic development and prosperity. Moreover, stunting’s long-term effects, including cognitive and learning impairments, limit a child’s ability to perform well in school, affecting individual potential and overall educational quality. Societies may face the perpetuation of poverty and inequality. Stunting is often more prevalent among marginalized and disadvantaged populations, exacerbating societal inequalities. It’s fundamentally a public health issue, straining healthcare systems with short-term health consequences like frequent infections and posing a broader public health challenge with increased risk of non-communicable diseases. Addressing stunting aligns with global development goals, particularly those related to health, education, and poverty reduction, emphasizing its critical role in global development and well-being.

## 3 Chrono-nutrition and health

Chrono-nutrition or circadian timing of food intake is a novel subject of study in which food influences the body’s circadian rhythm, including the distribution of frequency, regularity, and energy (28, 29). Significant chrono-nutrition behaviors include (1) skipping breakfast, (2) eating dinner, (3) eating the largest meal, (4) window eating, and (5) evening latency (30).

The circadian diet is important for the body’s metabolism and regulates food intake to ensure optimal health status. Unhealthy eating habits can affect a person’s behavioral, physical, and cognitive health. Circadian rhythm disturbances result in metabolic diseases or the development of morbidities, such as hypertension, dysglycemia, dyslipidemia, and abdominal obesity. Therefore, chrono-nutrition can affect one’s health in case of an unequal interaction between time and food intake (28).

In general, predictable environmental changes, such as temperature, light, diet, exercise, and noise, can be balanced by the presence of a circadian clock to promote optimal health. Almost all cells in the human body have self-contained molecular oscillators, which consist of clock genes, that drive the rhythmic expression of controlled genes for approximately 24 h. These genes make up 10–20% of the tissue transcriptome of circadian variation in enzyme activity, cellular activity, and hormone levels (31).

Maternal melatonin in the placenta trains the circadian rhythm of the fetus to transfer information during the day from mother to baby through breast milk, thereby helping the neonate to adjust to the external environment. This is possible due to circadian fluctuations in the composition of breast milk (31). Therefore, chrono-nutrition influences infant food intake and stunting. Newborns can obtain melatonin through breast milk, although only during night feeds. Melatonin levels in breast milk peak at midnight and 4 a.m. Exclusive breastfeeding has been shown to reduce colic and irritability in neonates. However, a lack of melatonin can occur in babies who are formula-fed or consume breast milk exclusively during the day (32).

Chrono-nutrition, with its focus on aligning dietary choices with the body’s biological clock and circadian rhythms, plays a significant role in preventing stunting. A clear relevance between chrono-nutrition and stunting prevention can be established based on its fundamental principles. First and foremost, chrono-nutrition emphasizes the timing of food intake, suggesting that meals should be consumed during daylight hours while discouraging late-evening eating. This approach ensures that children and pregnant women receive essential nutrients when their metabolic processes are most active and efficient (33). Such timely nutrient intake can optimize absorption and utilization, crucial for the healthy growth and development of children and thus reducing the risk of stunting.

In addition to timing, chrono-nutrition promotes nutrient-dense meals. Ensuring that children and expectant mothers receive the necessary vitamins and minerals for growth and development is at the core of stunting prevention. Furthermore, circadian disruptions may lead to metabolic and pregnancy disorders associated with stunting (34, 35). Chrono-nutrition can play a role in mitigating these disruptions by promoting the alignment of dietary choices with the body’s natural rhythms. This, in turn, can help reduce the risk of metabolic and pregnancy-related issues that contribute to stunting.

In summary, chrono-nutrition’s relevance to stunting prevention lies in its emphasis on the timing, quality, and diversity of dietary choices, which are essential for optimal nutrition in children and expectant mothers. By ensuring individuals receive the right nutrients at the right times, chrono-nutrition contributes to reducing the risk of stunting and supporting healthy growth and development in the early stages of life.

## 4 Current research on the prevention of stunting

Several risk factors, including FGR and premature birth, can result in stunting in early infants (7). Birth cohort analysis of data from 19 studies in low- and middle-income countries (LMICs) revealed that babies with FGR and premature birth were more likely to stunt later in life than babies born at full term and appropriate for gestational age (AGA) (36). The stages of growth in early life are strongly correlated with the mother’s height, reflecting both environmental and inherited factors. Childhood stunting contributes in part to adult short stature. Significant research conducted in Guatemala revealed that both the study participants and the supplemented group’s second-generation offspring, who were born with larger head circumferences and birth weights than their parents, benefited from early nutritional (folic acid, iron, zinc, and vitamin B12) supplementation (37). Another cohort study also found that short height is highly correlated with small for gestational age (SGA); the adjusted odds ratio for women ≤145 cm was 2.03 (95% CI 1.76 to 2.35) for term SGA in 12 cohort studies (38). Micronutrient deficiencies, weight gain during pregnancy, maternal body mass index (BMI), and moderate-to-severe anemia are additional nutritional factors that are associated with the risk of SGA births (39). Low-BMI women (<18.5 kg/m^2^) are at increased risk of delivering SGA babies, according to a study of eight cohorts (relative risk: 1.41 [1.24–1.60]) (7). Dietary counseling and the delivery of balanced calorie and protein supplements—which provide approximately 25% of the total energy as protein—have been used in pregnancy nutrition therapies. Protein is an essential nutrient for pregnant women who are malnourished. A systematic review found that taking energy protein balance supplements reduced SGA birth risk by 34%, with more pronounced effects in undernourished women (40).

Providing a baby with nutrient-dense meals in addition to breast milk begins around the age of 6 months and continues until the baby stops breastfeeding. Moving on from cohort studies, observational studies have reported a link between stunting and dietary factors. Survey results from seven Latin American countries revealed a strong correlation between supplementary feeding methods and height-for-age z-scores (41). In Indonesian community-based studies, increasing the amount of supplemental feeding per day had a favorable effect on growth (42), and in Bangladesh, high dietary diversity had a positive effect on growth (43). According to additional research utilizing data from demographic and health surveys, the stunting risk was reduced by following a diet that included timely solid food, dietary diversity, and iron-rich food in addition to the minimum acceptable diet by WHO (44). Education or counseling for caregivers on suitable complementary feeding practices or the provision of supplemental meals are examples of dietary interventions (45).

Supplementation has been the main intervention against stunting, and evidence is provided in Table 1. Table 1 presents several studies discussing methods to prevent stunting, such as administering lipid-based vitamin supplements, which have been shown to decrease the risk of stunting (46, 47). Most of the studies suggest that lipid supplements could increase length in children because lipid-based nutrient supplements (LNS) offer a variety of vitamins, protein, energy, minerals, and necessary fatty acids, unlike most other micronutrient supplements (49). Over the years, a wide range of LNS products have been developed. Fully prepared therapeutic meals (100 g; 2092 kJ [500 kcal]/serving) and large-quantity LNS products were developed to treat severe acute malnutrition. Medium-quantity LNS or prepared supplemental meals (40–50 g; 1,046 kJ [250 kcal]/serving) are employed in the nutritional therapy of severe and moderate acute malnutrition as they offer more than one-half of the daily energy requirement. Small-quantity LNS products (SQ-LNS) provide a lower energy dose (20 g; 460–628 kJ [110–150 kcal]/serving) and 50% of the required nutritional intake for micronutrients and essential fatty acids.

**Table 1:** Current research on the prevention of stunting.

No	Author, Year	Study design	Participants	Intervention	Outcomes	References
1	Muslihah et al., 2016	RCT	168 infants aged 6–12 months in the West Madura countryside	SQ-LNS	Throughout the 6-month intervention, there were substantially larger mean length gains (8.57 cm) and (changes in length-age-*Z* score) (0.09 unit).	Muslihah et al. (46)
2	Tam et al., 2020	Systemic review and meta-analysis of 197 studies	Children under the age of 5 years in low- and middle-income countries	Micronutrient supplementation (iron, iron & folic acid, multiple micronutrient (MMN), lipid-based nutrient (LNS), and micronutrient powder) and fortification	LNS can assist in reducing stunting and poor nutrition in children. The length-for-age *Z* scores were slightly enhanced by MMN supplementation.	Tam et al. (47)
3	Soofi et al., 2022	RCT based on a community cluster that was conducted in the Pakistani area of Rahim Yar Khan	197 children in Pakistan were divided into four clusters in which 434 children received UCT, 433 children received UCT and SBCC, 430 children received UCT and LNS, and 432 children received all three therapies	UCT, LNS, and behavior change	In 6–to 23-month-old infants, UCT coupled with LNS and UCT + LNS + SBCC were successful in decreasing the prevalence of stunting. LNS is crucial to preventing stunting because UCT and SBCC alone are ineffective in preventing stunting.	Soofi et al. (48)
4	Campos et al., 2020	Logistic regression model	2089 Mexican children	Breastfeeding	Breastfeeding is the primary preventive method for stunting. Low birth weight and consumption of complementary foods before 6 months of age are risk factors for stunting.	Campos et al. (54)
5	Leroy et al., 2018	Cluster randomized controlled study divided into four clusters; T18 (mothers and children who received interventions during gestation until the children were 18 months old), T24 (mothers and children received interventions during gestation until the children were 24 months old), TNFP (mothers and children received interventions from birth until the children were 24 months old), and control arm (got no interventions)	Stunted children in Burundi	Food-Assisted Integrated Health and Nutrition Program (Tubaramure)	The Tubaramure program included food ration distribution (received a corn-soy blend and vegetable oil fortified with micronutrients), enhanced health care delivery and advancement of the use of these services, and behavior change communication that had a significantly beneficial result on the T24, T18, and TNFP groups. Stunting continued to be common throughout the treatment group and worsened in the control group.	Leroy et al. (55)
6	Liu et al., 2018	Randomized controlled studies were systematically reviewed and meta-analyzed	Children younger than 5 years of age	Zinc supplementation	After labor, zinc supplementation improved height (WMD = 0.23 cm), weight (WMD = 0.14 kg), and weight-for-age *Z*-score (WMD = 0.04), but not height-for-age Z-score or weight-for-height *Z* score. Evidence indicated a greater benefit when supplements were given to children older than 2 years of age, and zinc supplementation in newborns and early childhood boosted expansion outcomes.	Liu et al. (51)
7	Adu Afarwuah, 2020	Randomized experiments were performed in Sub-Saharan Africa, where SQ-LNSs were given to children as young as 6 months and/or women who appeared to be in good condition	Moms and infants as young as 6 months of age	SQ-LNS	Compared with supplementation with micronutrients, prenatal SQ-LNS consumption decreased Ghana’s high prevalence of inadequate gestational weight gain.	Adu Afarwuah (56)
8	Dewey et al., 2021	Meta-analysis of 14 RCTs	Children aged 6–24 months	SQ-LNS	In contrast with the control group, in children who received SQ-LNS, stunting, wasting, and being underweight were 12–14% less common, and SQ-LNS consumption reduced the incidence of anemia by 16% and iron-deficiency anemia by 64%.	Dewey et al. (57)
9	Khan et al., 2020	Cluster RCT performed in Sindh’s Thatta and Sujawal districts. *n* = 870 (control group: 451; intervention group: 419)	Children aged 6–23 months in Pakistan	LNS	Children who received Wawamum (medium-quantity LNS) were less likely to experience stunting (RR = 0.91) and wasting (RR = 0.78).	Khan et al. (58)

For longer-term use and to address certain dietary deficiencies, SQ-LNS are better suited as home fortificants. They are used to combat undernutrition in more food-secure environments. Several factors impact the effect of dietary supplements, like SQ-LNS, on child growth, such as the target group’s characteristics, including baseline nutritional state, age, study location, income level, and methodology (adherence calculation, control group, duration of intervention) (50). SQ-LNS products supply less than one-half of the vital energy and are intended to avoid malnutrition and encourage growth and development (49). SQ-LNS (20 g) delivers 9.9 g of lipids per day or 12.3% of the daily calorie requirement (46, 47). In addition to LNS, zinc consumption after birth can increase a child’s height and weight. Zinc plays a role in cell growth, intestinal electrolyte absorption, neurotransmission, immunological response gene regulatory proteins, and hormonal receptors (51). Zinc fingers, which are significant domains associated with mRNA transcription, are created by the chelation of zinc with the amino acids cysteine and histidine. In addition, zinc modulates metallothionein gene expression, apoptosis, and synaptic signaling (52).

In addition to providing LNS, Soofi et al. outlined that unconditional cash transfer (UCT) and social and behavior change communication (SBCC) could be provided. UCT can improve nutrition by overcoming poverty so that the money can be used for preventive health care and increasing domestic meal consumption and food diversity. SBCC is an activity that encourages mothers and children to engage in healthy habits, namely regarding maternal nutrition, LNS use, and water, sanitation, and hygiene (WASH) (48). Breastfeeding is another method that can alleviate stunting in addition to vitamins. In LMICs, where the availability of clean, safe WASH systems is limited or non-existent, breastfeeding is essential as it protects children from non-harmful supplemental drinks and meals. The risk of diarrhea and other infectious disorders, which have previously been linked to stunting, may arise due to exposure to harmful drinks or foods (19, 53).

Current research on the prevention of stunting reveals several key findings. Stunting in early infants is associated with risk factors such as FGR and premature birth. Birth cohort analyses from LMICs indicate that babies with FGR and premature birth are more likely to experience stunting later in life. Maternal height strongly correlates with early childhood growth, reflecting both environmental and inherited factors. Nutritional supplementation during pregnancy and early life can have intergenerational benefits, improving birth weights and head circumferences in the second generation. Various nutritional factors, including micronutrient deficiencies, maternal body mass index (BMI), and anemia, are associated with the risk of SGA births. Dietary interventions and balanced calorie and protein supplements have been used to combat SGA.

Lipid-based vitamin supplements have been shown to decrease the risk of stunting, particularly in children, as they provide a wide range of essential nutrients. Zinc consumption has been linked to increased height and weight in children due to its role in cell growth and various physiological processes. UCT and SBCC can help improve nutrition by addressing poverty and encouraging healthy habits among mothers and children. Breastfeeding is an essential preventive method for stunting, particularly in areas with limited access to clean water and sanitation systems. In summary, current research on preventing stunting emphasizes the importance of nutritional supplementation, dietary interventions, lipid-based supplements, zinc, UCT, SBCC, and breastfeeding as effective strategies for reducing the risk of stunting in infants and children.

## 5 Potential implications of chrono-nutrition on stunting

Dr. Alain Delabos introduced the concept of chrono-nutrition in 1986, which is a dietary approach aligned with our biological clock, reflecting metabolic variations throughout the day. This method emphasizes the importance of meal timing, frequency, and regularity due to evidence associating late or irregular eating with increased risks of adiposity, Type 2 Diabetes (T2DM), and cardiometabolic factors. Chrono-nutrition, based on chronobiology, involves consuming the majority of calories and carbohydrates during lunch and early afternoon while avoiding late evening dinners. Beyond macronutrient and micronutrient composition, the timing of food intake during daylight hours as opposed to the evening or night plays a crucial role in maintaining metabolic health and potentially supporting weight management (59).

Chrono-nutrition is a fundamental concept that a person’s health is not only influenced by the quantity and quality of food but also by the timing of meals and the person’s circadian rhythm. In conjunction with the previous discussion, chrono-nutrition may contribute to stunting in pregnant mothers. Chrono-nutrition itself is not a cause of stunting. However, the concept of chrono-nutrition may indirectly contribute to preventing stunting by promoting healthier eating patterns, which can ensure that children and pregnant women receive essential nutrients at the right times for optimal growth and development. Changes in circadian rhythms, especially circadian clock gene expression, often occur in mothers during pregnancy. Circadian rhythms play an essential role in the function of specific metabolic genes during pregnancy, such as *PcK1*, G6Pase, and *GK* glucoregulation genes, to support a healthy pregnancy. If there is a disruption in the circadian rhythm, the pregnant woman may experience metabolic and pregnancy disorders, such as miscarriage, prematurity, low birth weight, and hypertension. For example, if a pregnant woman has a habit of staying up late or has a chronotype that motivates her to eat food at night, she has a higher risk of pregnancy disorders (60).

Mealtimes and the amount of carbohydrates consumed influence glucose metabolism. Energy intake by pregnant women at night, especially with poor diet quality and high carbohydrate and low fiber intake, can increase glucose tolerance and β-cell function during pregnancy and increase the risk of gestational diabetes mellitus. A second-trimester pregnant woman who eats late at night is at increased risk of giving birth prematurely (<37 weeks of gestation). This is because consuming food at night can suppress melatonin due to circadian misalignment, causing the dysregulation of oxytocin production, uterine contractility, and time of birth (60). Decreased melatonin levels during pregnancy have been shown to increase the risk of pre-eclampsia (61). Chrono-disruption is associated with stunting problems. Previous research suggests that premature babies are at increased risk of stunting compared with babies without problems during pregnancy (7, 36).

Fats, iron, tryptophan, melatonin, cholesterol, cortisone, and cortisol are strong indicators of the circadian variation in the composition of human milk according to a systematic review on circadian variation in human milk composition. Furthermore, there is convincing evidence that the total protein and carbohydrate content of human milk does not change over time (31). The available evidence for other components of human milk is insufficient to draw similar conclusions. Hypertension, infection, age, nutritional deficiencies, like iron deficiency, hypertension, and the length of time a mother has been lactating are maternal characteristics that can affect the circadian variation in human milk composition (62).

## 6 Conclusion

Stunting can be prevented by optimizing TF1000DL (63), starting by meeting nutritional needs from pregnancy until a child is 24 months old. In TF1000DL, optimizing the provision of nutrients supports the development of the fetus, maintains maternal health in preparation for the puerperium and breastfeeding, and helps prepare infants and toddlers to grow and develop optimally. Chrono-nutrition during breastfeeding is an important strategy to promote health and cognitive and behavioral neurodevelopment in infants (64).

On the other hand, circadian rhythms affect the success of pregnancy and optimal fetal growth (65). If there is a disturbance in the circadian rhythm, a pregnant woman may experience metabolic and pregnancy disorders, such as depression, prematurity, low birth weight, and hypertension (60). Eating at night can suppress melatonin due to circadian misalignment, affecting uterine contractility, oxytocin dysregulation, and time of birth. Maternal characteristics that can affect the circadian variation in human milk composition include age, hypertension, infection, malnutrition, duration of breastfeeding, and iron deficiency (62).

The fetus will follow the mother’s circadian cycle of temperature, metabolites, and hormones during pregnancy. Therefore, as preterm newborns are more likely to experience stunting, prevention of stunting starts with pregnant mothers. Circadian rhythm alterations affect gene expression and metabolism in pregnant women. Chrono-nutrition assists in aligning nutrition with the biological clock to ensure that the intake is appropriate. Therefore, it is recommended that newborns only consume breast milk after delivery because it contains chrono-biotic chemicals that help to establish the baby’s sleep cycle and result in better sleep metrics compared with babies who only consume formula (64). In light of the clear importance of breastmilk and feeding, it is essential for public places, such as workplaces, to have dedicated places for mothers to properly breastfeed their babies.

Based on the findings, proactive measures to address the issue of stunting and advocate for the incorporation of chrono-nutrition principles into our healthcare and nutrition initiatives are needed. The optimization of nutritional intake during the period spanning from pregnancy through the initial 24 months of a child’s life, coupled with the synchronization of dietary selections with circadian rhythms, can significantly contribute to the prevention of stunting and the promotion of robust fetal development. It is imperative to recognize that disturbances in circadian rhythms may result in severe health complications, including premature births and stunting. Therefore, a paramount focus should be placed on maternal well-being, ensuring the provision of appropriate nutrition, and fostering a harmonious relationship between breastfeeding practices and the inherent biological clock, all aimed at guaranteeing an auspicious commencement to the lives of our offspring. Furthermore, the establishment of supportive environments for breastfeeding, including dedicated spaces within workplaces, is of paramount importance, as it plays a pivotal role in safeguarding infant health.

## Data availability statement

The original contributions presented in the study are included in the article/supplementary material, further inquiries can be directed to the corresponding author/s.

## Author contributions

NT: Conceptualization, Formal analysis, Methodology, Supervision, Validation, Writing – original draft, Writing – review & editing. SF: Data curation, Formal analysis, Investigation, Writing – original draft. WG: Formal analysis, Software, Visualization, Writing – original draft. AA: Data curation, Writing – original draft. RB: Data curation, Formal analysis, Writing – review & editing. LR: Data curation, Investigation, Writing – original draft. AA: Software, Writing – review & editing. HB: Data curation, Writing – review & editing. MS: Formal analysis, Writing – review & editing. NM: Supervision, Validation, Writing – review & editing. HH: Supervision, Validation, Writing – review & editing. FN: Conceptualization, Formal analysis, Methodology, Software, Visualization, Writing – original draft, Writing – review & editing. BK: Supervision, Writing – review & editing. ES: Formal analysis, Writing – review & editing.
